# Facilitators and inhibitors of attitude and word-of-mouth intention toward adoption of digital municipal service systems: A stimulus-organism-response approach

**DOI:** 10.1371/journal.pone.0315009

**Published:** 2024-12-18

**Authors:** Md. Shamim Talukder, Quazi Tafsirul Islam, Ziaul Karim

**Affiliations:** 1 Department of Management, School of Business and Economics, North South University, Dhaka, Bangladesh; 2 School of Business and Economics, United International University, Dhaka, Bangladesh; Taylor’s University - Lakeside Campus: Taylor’s University, MALAYSIA

## Abstract

Increased technology adoption has significantly transformed how governments interact with citizens. Today, e-government services and tools are integral to modern public administration. Factors affecting users’ adoption of e-government services have been studied in the past. However, this study focuses on citizens’ acceptance and resistance to such services, which have not been thoroughly explored. This study addresses the gap by examining the facilitators and inhibitors affecting users’ perceptions of the Digital Municipal Service System (DMSS). An integrated research model was developed based on the Stimulus-Organism-Response (SOR) framework. Using Smart-PLS, the study validated 353 respondents’ data collected from Bangladesh. The study revealed that platform quality, convenience, social, and inclusiveness values significantly influenced attitudes toward DMSS adoption. On the other hand, tradition and usage barriers significantly negatively impacted attitudes toward DMSS adoption. These findings offer important insights for policymakers involved in developing and implementing e-government services in emerging economies such as Bangladesh. The study also provides a foundation for further research on technology adoption.

## 1. Introduction

In today’s digital age, e-government services have emerged as an essential mechanism for governments to deliver efficient and effective services to citizens. Public service digitalization has improved significantly in terms of quality and accessibility, which has led to improved citizen-government interactions [1]. Digitizing government services has several benefits, as it reduces cost, enhances service delivery speed and decreases administrative costs, and allows citizens to interact with government services using a single tool [2, 3]. Digital Municipal Service System (DMSS) has been introduced as a one-stop platform aimed at improving citizen-government interactions in Bangladesh. However, as with many other e-government platforms in developing nations [4, 5], the success of DMSS is dependent on the willingness and enthusiasm of citizens to adopt the system. Even though DMSS has the potential to improve the delivery of government services, there is a lack of knowledge about why some citizens are inclined to adopt and use DMSS, whereas others are unwilling to do so.

While E-government service delivery platforms continue to evolve and expand, Information systems (IS) researchers have consistently explored how citizens perceive e-government services. It has been argued that previous studies have only focused on the factors that promote the adoption or usage, known as enablers while ignoring the factors that hinder adoption. Numerous factors have been identified in earlier research as enablers of e-government service adoption, such as perceived usefulness, perceived ease of use, trust in government, technological infrastructure, social influence, and citizen awareness of the services [1, 6–10]. Despite the focus on enablers, understanding the inhibitors is equally crucial for comprehensive insights into e-government adoption. Factors such as low digital literacy, privacy and security concerns, lack of trust in online platforms, inadequate infrastructure, and cultural resistance often act as significant barriers to adoption [11–13]. Some studies in recent years have shown data privacy and the complexity of using digital platforms deter citizens in emerging economies from fully embracing e-government services [14]. Besides, socio-economic factors, such as income disparities and access to reliable internet, are identified as factors that widen the digital divide [15]. This makes e-government platforms less accessible to certain segments of the population.

In order to understand the acceptance and resistance factors in e-government adoption from both theoretical and practical perspectives, it is important to study facilitators and inhibitors together. Theoretically, existing models such as the Technology Acceptance Model (TAM) and the Theory of Reasoned Action (TRA) emphasize facilitators that explain why citizens may adopt e-government services [8]. However, these models are inadequate as they do not account for inhibitor or resistance factors such as privacy concerns, digital literacy gaps, and trust issues [16]. In reality, focusing solely on facilitators risks creating policies that neglect critical barriers, thereby limiting the success of real-world implementation. Considering both enablers and inhibitors allows governments to design more inclusive e-government systems. This helps address the diverse needs of people while mitigating factors that contribute to resistance [17]. Policies that consider both viewpoints are crucial for increasing adoption rates and ensuring the long-term success of e-government platforms. Only then can we develop a model combining facilitators and barriers into a unified framework.

This study intends to investigate the facilitators and inhibitors of attitude and word-of-mouth intention toward adopting DMSS. The following research questions are proposed to fulfill the research objective:

- What are the critical factors influencing citizens’ attitudes and word-of-mouth toward intentions to use DMSS, and how are these factors related to enablers and inhibitors? and
- How can this integrated aspect be used to draw users toward e-government services in Bangladesh and other developing countries?

This study used the stimulus-organism-response (S-O-R) paradigm proposed by Mehrabian and Russell [18] to investigate the research questions. The S-O-R framework integrates concepts from consumer behavior, information systems, and psychology to understand the significance of DMSS in influencing users’ attitudes and word-of-mouth (WOM) intentions. Attitude represents an individual’s positive or negative evaluation of a technology or service, which can shape their intention to adopt or use it [19]. As e-government services are voluntary, users formed their intentions to use the system primarily based on their attitude toward using the system [20]. Thus, this study highlights the crucial role of attitude in predicting the intention to use DMSS, given that its use is voluntary. Besides, consumers’ positive perception was regarded as an enabler, and negative perception was considered an inhibitor, similar to the dual-factor model of Herzberg’s Two Factor Theory [21]. We also applied the theory of consumption values (TCV) [22] as enablers and the innovation resistance theory (IRT) as an inhibitor (S). Our research suggests that enablers drive the adoption of DMSS, but inhibitors may hinder their usage, which acts as the organism (O). The study evaluates user organismic states and WOM as a response (R) to the stimuli.

WOM plays a crucial role in engaging a larger number of citizens and fostering trust in government initiatives [23]. As citizens increasingly share their opinions and experiences with e-governance services across various social platforms, WOM serves to increase citizen engagement and interactions with governments [24]. WOM provides citizens with information, enabling their engagement with government entities and influencing their future behavior toward government services [25, 26]. So, the application of WOM in this study helps extend the existing e-government body of literature. By using the S-O-R framework, this research aims to provide a complete understanding of the factors shaping citizens’ attitudes toward using DMSS. It offers insights on encouraging the use of e-government services in developing countries. Thus, this research is essential in fully comprehending the facilitator’s and inhibitors’ perspectives of e-government systems.

## 2. Background literature

### 2.1. E-government acceptance and resistance

E-government adoption is complex because it involves multiple stakeholders with different and sometimes conflicting interests [27]. Citizens, government bodies, and service providers have changing expectations and needs as technology and user demands also evolve. This makes it difficult to adopt a single solution, as stakeholders must put a combined effort to adjust to these shifting demands [28]. As new e-government platforms are introduced, existing theories can barely explain the complexity of consumer acceptance. The rapid advancement of technology has created a landscape where user expectations change frequently, highlighting continuous adjustments to electronic services and delivery methods [17]. This constantly changing environment underlines the need for developing novel theoretical frameworks to better understand and predict e-government service adoption, especially since existing frameworks do not always account for this variability [29].

The majority of the existing literature on e-government adoption focuses on enablers that drive user acceptance and applied well-established theoretical frameworks (e.g., UTAUT, TAM, SCT, IS success model) [8, 20, 30]. These studies mainly emphasize the role of perceived usefulness, ease of use, social influence, and trust in shaping users’ intentions to adopt e-government services. Despite the extensive focus on enablers, a smaller body of research has attempted to explore the barriers or inhibitors to e-government adoption [11–13, 31]. Factors such as digital literacy gaps, privacy concerns, lack of trust, and resistance to change have been found in distinct studies as critical barriers in e-government adoption research. However, these studies often lack a structured theoretical model (e.g., innovation resistance theory, status quo bias, decision avoidance theory) to adequately capture the full complexity of inhibitors. Moreover, a key limitation in current research is the lack of studies that examine enablers and inhibitors simultaneously. This has left a significant gap in understanding how these two opposing forces interact and shape user adoption decisions in real-world e-government implementations. Therefore, this research should aim to bridge this gap by developing integrated models that simultaneously consider facilitators and barriers, providing a more comprehensive understanding of e-government adoption dynamics.

### 2.2. The stimulus-organism-response (S-O-R) model

Environmental psychology literature often applied the S-O-R paradigm, first introduced in 1974 by Mehrabian and Russell. The framework suggests that environmental cues (stimuli) perceived by individuals can prompt internal evaluative processes (organism), leading to either positive or negative behaviors (response) toward the stimuli [18, 32]. The stimulus is the input that prompts the organism to act, such as by launching a new e-government service, when it comes to adopting e-government services. The person who is exposed to the stimuli and can respond to it, in this situation, the user’s attitude, is the organism. The organism’s output is the response, which might take the form of behavioral, emotional, or cognitive reactions [33, 34].

The S-O-R model highlights the importance of understanding how the stimulus, organism, and response interact when studying citizens’ acceptance of e-government services. The model has been successfully utilized to understand consumer innovation adoption in earlier literature.

The model was used by Tak and Gupta [35] to explore the intentions to use the travel app and Yuan to examine the adoption of mobile payment loyalty. Chopdar and Balakrishnan [36] employed the model to investigate the repurchase intention and satisfaction experience of Indian consumers in m-commerce. These studies show the ongoing relevance of the S-O-R model in examining consumer behavior. Therefore, the S-O-R model is a strong foundation for our research as it helps examine different factors specific to the application and their overall effect on user behavior.

### 2.3. Dual factor theory (DFT)

The dual-factor model explains that both positive and negative factors influence how quickly consumers accept or reject a product or service [31]. Positive factors, called facilitators or enablers, encourage greater use of the product or service [37]. For example, the usefulness of an e-government platform may lead to a higher adoption rate, while a lack of usefulness may discourage usage. On the other hand, some factors may be uniquely negative and discourage usage without being simply the opposite of an enabler. These factors are known as inhibitors and only discourage usage [37]. For instance, if paying an electricity bill goes smoothly, it will be viewed positively. Still, if the process is hindered by negative perceptions such as transaction risk, it may discourage the usage of the e-government system.

In this study, we choose the enabler-inhibitor viewpoint for four reasons. Firstly, prior IS research has mainly highlighted the enablers, with limited consideration for inhibitors [38]. Secondly, adverse events tend to be more noticeable and significant than positive events, implying that inhibitors may significantly affect user adoption more than enablers [39]. Thirdly, inhibitors may be more evident due to their harmful nature, potentially overwhelming the positive impact of enablers. Lastly, by analyzing enablers and inhibitors, we aim to uncover the unique factors affecting the use of e-government services and provide recommendations for promoting and hindering the usage.

## 3. Research model and hypothesis development

### 3.1. Theory of consumption values as enablers

As previously mentioned, enablers are positive factors that promote or encourage using a product or service [40]. The Theory of Consumption Values (TCV) explains how different factors shape consumer behavior, such as adopting e-government systems. It has three key ideas: people’s actions are influenced by various values, the importance of these values changes based on the situation, and these values work independently of each other [22].

The TCV framework acknowledges that contextual or situational factors can influence consumer behavior rather than focusing solely on predefined factors [16, 41, 42]. Therefore, understanding the usage context is vital for implementing the TCV successfully. In this study, we build upon the TCV’s categorization of consumption values and apply it to our context of DMSS adoption. We identify six dimensions of value that may facilitate citizens’ use of the DMSS platform: quality of the platform, convenience value, social value, inclusivity value, conditional value, and epistemic value. This expanded framework allows for a more comprehensive understanding of the various factors contributing to adopting DMSS.

Functional value refers to the usefulness of a product or service based on how well it performs specific tasks, such as being durable and reliable [22]. In e-government platforms, functional value is reflected in two key areas: platform quality and convenience. Platform quality is judged by how durable and reliable it is, ensuring it provides timely information and services that efficiently meet citizens’ needs. The quality and convenience of e-government platforms significantly influence citizens’ attitudes and intentions to use such services [10]. Past studies have shown that quality is essential to functional value [41]. Convenience, in this context, refers to how easy the platform is to access, navigate, and use and how quickly citizens can access services. Talwar, Dhir [43] highlighted that convenience plays a significant role in shaping consumer behavior and improving satisfaction. For DMSS, the convenience value is vital because citizens can use the platform to complete tasks like paying taxes, getting certificates, paying bills, and managing properties.

Functional value is an essential aspect of e-government adoption, and prior studies have demonstrated the significance of perceived quality and convenience in shaping users’ attitudes toward services or products across various contexts [41, 44]. This could lead to a positive attitude toward using the DMSS for their e-government needs. We assume the following hypotheses based on the preceding discussion.

*H1*. *The quality of the DMSS platform positively impacts citizens’ attitudes*.
*H2*. *Convenience value has a positive impact on citizen’s attitudes toward DMSS*.

The term "social value" describes how much utilizing a product or service improves a user’s connections, identity, or standing in society [22]. This study utilizes two concepts, social image, and inclusiveness value, to capture the dimensions of social value. First, the use of new technology, such as DMSS, can lead to an increase in social status and image, as they are considered trendy in developing countries. Earlier research has shown that social image can impact individuals’ behavioral intentions to use a specific IS [10, 45, 46].

E-government platforms can provide all citizens’ services regardless of income or education level [10]. This way, e-government platforms can provide public services equitably and increase accessibility for all citizens. Research supports the notion that inclusiveness in e-government systems positively impacts citizens’ attitudes toward Digital Municipal Service Systems (DMSS). The inclusiveness value is tied to individuals’ perceptions that e-government platforms make public services more accessible and delivery more transparent, fostering positive attitudes Hornung and Baranauskas [47]. Studies by Al-Hujran et al. [20] confirm that public value, ease of use, and demographic factors enhance inclusiveness, thereby influencing citizen engagement. A recent study by Li and Shang [10] found that inclusiveness value is a significant dimension of an e-government system’s overall value and plays a vital role in the broader adoption of public services. Therefore, it is suggested that the inclusiveness value will result in a positive attitude toward using the DMSS. As a result, the following hypotheses are proposed:

*H3*. *Social value has a positive impact on citizen’s attitudes toward DMSS*.
*H4*. *Inclusiveness value has a positive impact on citizen’s attitudes toward DMSS*.

The conditional value represents how useful a service or product is in specific situations [22]. Wang, Liao [48] further describe how the value of a good or service is directly linked to its relevance in specific situations. This can be the temporary functional or social value that arises from a need created by the circumstances [22]. This creates a positive attitude towards using the platform, as supported by the work of Savoldelli, Codagnone [49], who illustrate how the perceived usefulness in specific contexts influences adoption behavior.

The government can promote platform use by offering discounts on services compared to traditional methods. For example, e-filing taxes is cheaper than manual submission. When citizens find services on the platform at a lower cost, this creates conditional value, which is expected to encourage a positive attitude toward using DMSS. As a result, the following hypotheses are proposed:

*H5*. *Conditional value has a positive impact on citizen’s attitudes toward DMSS*.

The perceived benefit from gaining knowledge or information through goods or services is called epistemic value [22]. According to prior literature, acquiring knowledge can increase self-efficacy and self-esteem. This increase can lead to positive attitudes toward using a product or service [17, 50]. In the context of DMSS, providing knowledge and information through these services can lead to a positive attitude towards their usage. For instance, if citizens feel that DMSS will provide them with relevant and trustworthy information on government policies, services, and procedures, they may be more likely to use it [10, 47]. Additionally, accessing and using e-government services can be viewed as a means of gaining knowledge and empowerment, which can also lead to positive attitudes towards their usage [17, 44]. Based on existing research, we assume that epistemic value can positively affect user attitudes towards using DMSS. Therefore, we propose the following hypothesis:

*H6*. *Epistemic value has a positive impact on citizens’ attitudes toward DMSS*.

### 3.2. Innovation resistance theory (IRT) as inhibitors

The IRT suggests that various factors may lead to consumers’ resistance to using an innovation [51, 52]. According to Kushwah, Dhir [53], it is identified that there are two significant aspects of consumer resistance. They are functional barriers and psychological barriers. Changes in consumption patterns cause functional obstacles. On the other hand, conflicts between consumers’ beliefs and particular products cause psychological barriers. The most common functional barriers are related to usage, risk, and values. At the same time, psychological barriers are related to image and tradition. This study identifies the risks, usage, and traditional barriers preventing consumers from embracing a new system such as DMSS.

A tradition barrier is a reluctance to adopt new technologies because of a preference for familiar practices and cultural norms [54]. Polites and Karahanna [55] identified that users often persist with an existing system despite incentives and better outcomes associated with change. Hoque and Sorwar [56] suggest that past behavior influences people’s decisions. It makes them overestimate the benefits of new systems. This bias reduces their engagement with new technology and lowers their intention to use it. Those with a tradition barrier tend to rely on past behavior and limit the information they consider [17]. In this context, citizens may visit local government offices physically instead of using the recent DMSS, resulting in lower intentions to use the platform. Based on these arguments, we propose the following hypothesis.

*H7*. *Tradition barrier has a negative impact on citizens’ attitudes toward DMSS*.

Perceived risk is the subjective assessment of potential adverse outcomes or uncertainties associated with a decision [57]. According to Saxena [58], data security, privacy, lack of control over personal information, and the threat of online fraud can influence the adoption of e-government services. These concerns often increase the perceived risk, leading to a negative attitude among users and reducing their likelihood of using such services. Research has shown that around 80% of internet users express concerns about revealing their identities online [59]. This highlights how risk perception can be a significant barrier to adopting and using electronic services [17]. Thus, individuals may hesitate to use DMSS due to concerns about the safety and confidentiality of their personal information. Based on this argument, we hypothesize that;

*H8*. *Risk barrier has a negative impact on citizens’ attitudes toward DMSS*.

The term "usage barrier" refers to the incompatibility of consumers modifying their usual behaviors, habits, and routines to adopt an innovation [60]. This concept suggests that the usage barrier manifests when customers must adjust their typical usage patterns to accommodate a new product or service, requiring effort and adaptation. In e-government services, these barriers often involve a lack of technical skills, limited technological access, and complex website designs. These challenges and lack of self-confidence can deter adoption and halt the effective use of technology [44].

Research shows that usage barriers negatively impact users’ behavioral intentions toward adopting new technologies. For example, barriers like unfamiliarity or complexity hinder individuals from embracing new technologies [10, 61]. In the context of mobile payment services, Kaur, Dhir [52] found that the usage barrier was a crucial factor influencing whether individuals adopted these services. Also, recent studies confirm that usage barriers, such as limited computer literacy and high internet access costs, pose significant challenges to e-government adoption, particularly in developing nations [44]. Based on this argument, we hypothesize that;

*H9*. *Usage barrier has a negative impact on citizens’ attitudes toward DMSS*.

According to the S-O-R framework, response (R) represents the final behavioral outcome of the organism [18]. In the context of this study, the response variable examined was word of mouth (WOM). Attitude towards DMSS is the cognitive state (O) discussed in this study. Attitude is a learned, persistent, and evaluative mental and neural state. It shapes an individual’s feelings, beliefs, and behavioral intentions toward a specific object, person, or event. This attitude impacts psychological and behavioral responses [62, 63]. Likewise, WOM refers to informal communication about products, services, or brands that occurs between consumers without the direct involvement of the company or brand [64]. It is considered one of the most powerful marketing tools as it is based on personal experiences and opinions, which can significantly influence the purchasing decisions of other consumers. Previous studies have shown that positive WOM can enhance individuals’ perceptions of the value and simplicity of e-government services. As a result, their attitude towards the technology becomes more positive [65]. WOM is a powerful informal communication tool that can persuade users to make decisions by providing others’ experiences and recommendations about a product or service [66]. It is incredibly influential for individuals who perceive high risks and have limited information about their choice of a service or a product. Therefore, WOM is crucial in determining users’ acceptance of DMSS. Thus, we propose the following hypothesis:

*H10*. *A positive user attitude towards DMSS leads to an increase in the intention to spread word-of-mouth (WOM) about the system*.

**Fig 1:**
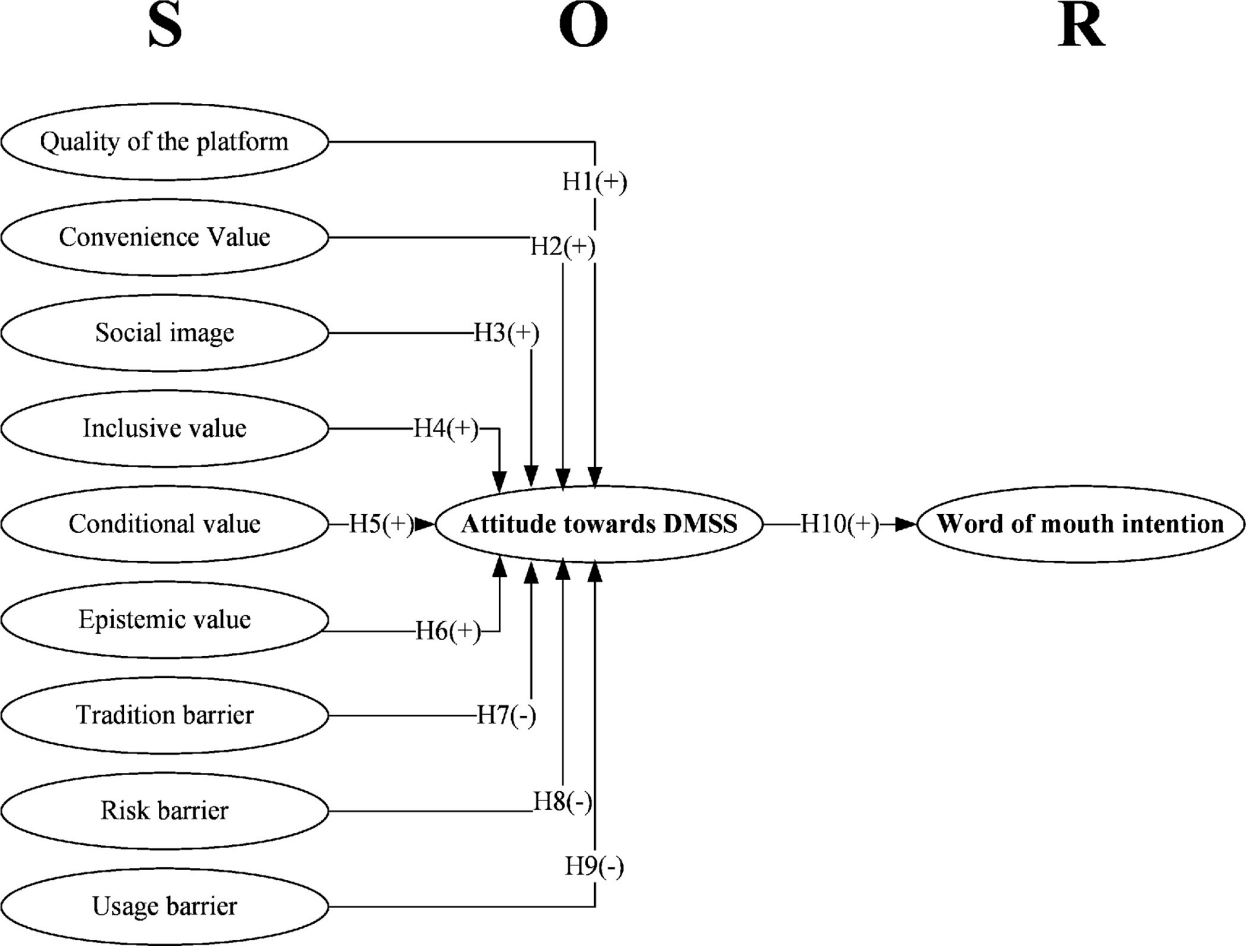
Proposed research model.

Based on the above discussion and proposed hypotheses, the research model is presented with their hypothetical relationships in Fig 1 below.

## 4. Methodology

### 4.1. Study context

The digital municipality services system (DMSS) project, launched in 2019, automated five citizen services, including councillor’s certificate, property management, trade licenses, holding tax, and water billing services. Korea International Cooperation Agency (KOICA) plans to grant $8.5 million to expand the pilot to all 329 municipalities in Bangladesh. The project, running from 2022 to 2026, will establish the national municipality digital service system, upgrade and expand the existing municipality services by providing equipment, and strengthening the capacity of Bangladeshi officers. The platform aims to simplify and streamline the process of accessing government services, making it easier and more convenient for citizens. DMSS was introduced to address the challenges faced by citizens in accessing traditional government services, which were often time-consuming, bureaucratic, and non-transparent. However, e-government initiatives, including DMSS, usually fail in developing countries due to the disparity between the idealized design and the practical implementation of e-government systems [20, 44]. The Bangladeshi government must consider the societal, technological, and security factors that impact the success of e-government in their specific context. This consideration will help bridge the gap between the envisioned e-government design and its implementation. In the context of Bangladesh, understanding the factors that influence citizen adoption of e-government services is of paramount importance for policymakers. This knowledge can inform the design of e-government services, enhance service delivery processes for public organizations, and attract and retain users for government agencies.

### 4.2. Measurement of constructs

The research team used existing measurement items from previous studies, which they adapted to the specific context of DMSS. Each statement describing every single item was measured on a 7-point Likert scale ranging from 1 –“Strongly disagree” to 7 –“Strongly agree”. Participants were asked to respond to a series of statements by indicating their level of agreement. In S1 Appendix, the measuring items are listed. Among the two survey sections, the first section contained 37 items associated with the 11 constructs in the proposed research model. The second section of the survey questionnaire asked about the participant’s gender, age, education, experience, and region. According to the guideline by Rahi [67], a limited-scale pre-test involving 20 graduate students was conducted to refine our survey questionnaire. Based on their valuable feedback, we slightly adjusted the questionnaire’s structure and wording, enhancing its clarity and relevance for the subsequent study.

### 4.3. Sample selection and survey method

The study focused on Bangladeshi citizens who had prior experience using the DMSS. The ethics committee has confirmed that no formal ethical approval was required because the data is anonymous, not sensitive or confidential, and no vulnerable or dependent groups were included. This study has been performed by the Declaration of Helsinki. The research team used non-probability convenience sampling; a method commonly used in studies analyzing the usage of e-government services [7, 17, 68]. This type of sampling allows for efficient data collection, improved data quality, and increased control over the sample selection process [69]. Even though it is not typical of the population, it allows the researcher to gather a representative sample of the population of interest, leading to more excellent knowledge and insightful discoveries [70].

Recruiting participants in the study involved approaching individuals at different strategic locations such as parks, marketplaces, and local government offices. Participants who agreed to participate were given a comprehensive explanation of the purpose of the study and their rights as participants, including the provision of an informed written consent form. The participants were then notified of the data collection method involving a survey. To ensure the confidentiality of the participants’ information, the researcher explained the measures that would be taken, such as using coding the data. Participants were given information about the anticipated time needed to complete the survey and were offered a token of appreciation for their participation. This approach enhanced participant engagement and facilitated a smoother data collection process [71]. The data collection for this study took place over a period of three months, from April 2021 to June 2021.

The sample size was determined based on standard guidelines for SEM, which recommend a minimum of 200–300 responses for valid model estimation [72]. We also used G*Power software to determine the minimum sample size requirement [73]. Based on the analysis, a minimum of 88 participants was required to achieve an acceptable level of statistical power for detecting significant relationships between variables. Therefore, we plan to collect more than 300 sample to satisfy the minimum sample size requirement for this study. We distributed 380 physical copies of the questionnaire to the respondents who agreed to participate in the survey. All the participants completed the survey during the data collection process. To minimize statistical biases due to missing data, 27 responses were excluded from the analysis as they contained more than 10% missing responses across all items, following the guidelines outlined by Joseph, Black [74]. Eliminating responses with excessive missing data helped ensure the results’ validity and reliability. This led to a final sample of 353 usable responses.

### 4.4. Data analysis

The proposed model was analyzed using partial least square structural equation modeling (PLS-SEM), the analytical approach recommended for analyzing a complex path model [63]. We conducted the PLS-SEM analysis using SmartPLS version 3, a widely employed theory testing and validation tool. PLS assesses psychometric properties and offers substantial evidence regarding the presence or absence of relationships [75]. Notably, it delivers more dependable results when dealing with non-normally distributed data and small sample sizes compared to component-based SEM [76]. In this study, we followed a two-stage methodology outlined by Anderson and Gerbing [77]. In the first stage, we tested reliability and validity tests using a measurement model, and secondly, we assessed the structural model to validate the hypotheses.

### 4.5. Demographics

Gender distribution shows a relatively balanced representation, with 53.54% male and 46.46% female respondents. In terms of age distribution, the majority of respondents are between 18 and 35 years old (63.73%), while smaller percentages fall into older age brackets. When it comes to education, a significant proportion of respondents hold Bachelor’s degrees (58.07%), followed by Master’s degrees (34.84%). The data also highlight respondents’ experience with e-government services, with a substantial portion indicating usage of less than a year (41.93%), followed by 1–2 years (40.51%). Geographically, the majority of respondents reside in urban areas (53.54%), while the remaining respondents live in semi-urban areas. It is worth noting that there are no respondents from rural areas in our sample, as the DMSS platform is only available in selected urban and semi-urban areas. An outline of the respondents’ profiles can be found in S2 Appendix.

## 5. Results

### 5.1. Common method bias

This study applied several solutions to limit common method bias concerns [78]. Firstly, the respondents’ personal information is kept confidential, and the order of questions is changed to avoid revealing the research structure. Second, Harman’s single-factor test was conducted to evaluate the likelihood of the standard bias method occurring. The analysis results of Harman’s single-factor test showed that the single factor explained 36.45 (<50%) of the total variance, so the common method bias did not seem to occur in this study [79]. Moreover, the variance inflation factor (VIF) values have been assessed for collinearity analysis between the indicators before undertaking the research model path analysis. The multicollinearity problem may have affected the model if the VIF values are equal to or greater than 3.3 [72, 80]. According to Table 1, the indicators’ VIF values are below 3.3, and the collinearity between the indicators employed in the research model study has been considered without any problems.

**Table 1:** Measurement model.

Constructs	Items	Loadings	VIF	Cronbach’s Alpha	CR	AVE
Attitude	ATT1	0.751	1.494	0.790	0.864	0.615
	ATT2	0.745	1.494			
	ATT3	0.872	2.036			
	ATT4	0.762	1.541			
Convenience value	COV1	0.874	1.919	0.850	0.909	0.768
	COV2	0.870	2.183			
	COV3	0.884	2.156			
Conditional value	CV1	0.779	1.381	0.714	0.840	0.636
	CV2	0.822	1.459			
	CV3	0.792	1.369			
Epistemic value	EPV1	0.878	1.948	0.836	0.901	0.753
	EPV2	0.869	2.054			
	EPV3	0.856	1.871			
Inclusiveness value	INV1	0.873	2.195	0.881	0.926	0.807
	INV2	0.915	2.694			
	INV3	0.906	2.597			
Platform quality	PQ1	0.778	1.453	0.777	0.855	0.597
	PQ2	0.722	1.461			
	PQ3	0.833	1.650			
	PQ4	0.754	1.541			
Risk Barrier	RB1	0.977	2.077	0.861	0.874	0.702
	RB2	0.716	2.318			
	RB3	0.798	2.216			
Social Value	SV1	0.865	1.876	0.849	0.909	0.768
	SV2	0.874	2.155			
	SV3	0.890	2.269			
Tradition Barrier	TB1	0.848	1.951	0.835	0.890	0.669
	TB2	0.825	1.922			
	TB3	0.773	1.636			
	TB4	0.824	1.822			
Usage Barrier	UB1	0.759	1.421	0.795	0.865	0.617
	UB2	0.832	1.677			
	UB3	0.722	1.751			
	UB4	0.824	2.143			
Word of Mouth	WOM1	0.919	2.864	0.832	0.898	0.748
	WOM2	0.934	2.974			
	WOM3	0.727	1.466			

Note: CR = Composite Reliability, AVE = Average variance extracted

### 5.2. Measurement model assessment

The measurement model was evaluated using convergent validity, indicator reliability, internal consistency, and discriminant validity criteria. As shown in Table 1, factor loadings, which represent the correlations between observed variables and their underlying constructs, ranged from 0.716 to 0.977, all exceeding the recommended threshold of 0.7, indicating strong indicator reliability. The internal consistency of each construct was measured using Cronbach’s alpha (α) and Composite Reliability (CR). α values ranged from 0.714 to 0.881, while CR values ranged from 0.840 to 0.926, both surpassing the minimum acceptable threshold of 0.7, confirming the reliability of the constructs. Average Variance Extracted (AVE) values ranged from 0.597 to 0.807, exceeding the required threshold of 0.5, thus demonstrating convergent validity [72].

Discriminant validity, which ensures that constructs are distinct from one another, was assessed using two methods. First, according to Fornell and Larcker [75] criterion, the square roots of the AVE values for each construct were higher than the correlations between constructs (Table 2). Second, the Heterotrait-Monotrait (HTMT) ratio of correlations was calculated, with all values below the 0.90 threshold (Table 3), meeting the discriminant validity criteria [81].

**Table 2:** The correlation matrix and the square root of the AVE.

Constructs	Mean	SD	ATT	COV	CV	EPV	INV	PQ	RB	SV	TB	UB	WOM
**ATT**	5.25	1.42	0.78										
**COV**	4.88	1.37	0.71	0.88									
**CV**	4.95	1.39	0.49	0.41	0.80								
**EPV**	4.45	1.54	0.63	0.59	0.34	0.87							
**INV**	4.84	1.38	0.73	0.74	0.40	0.68	0.90						
**PQ**	3.63	1.47	0.60	0.50	0.30	0.60	0.61	0.77					
**RB**	3.16	1.04	-0.05	-0.03	-0.06	-0.08	-0.05	-0.05	0.84				
**SV**	5.14	1.33	0.71	0.56	0.36	0.62	0.63	0.50	-0.04	0.88			
**TB**	3.09	1.27	-0.71	-0.60	-0.39	-0.56	-0.63	-0.51	0.05	-0.68	0.82		
**UB**	2.81	1.22	-0.26	-0.16	-0.16	-0.16	-0.16	-0.19	-0.15	-0.13	0.22	0.79	
**WOM**	5.32	1.21	0.57	0.50	0.29	0.46	0.51	0.40	0.08	0.51	-0.56	-0.18	0.87

Note: ATT stands for Attitude, SV for Social Value, EV for Emotional Value, EPV for Epistemic value, COV for Convenience Value, INV for Inclusiveness Value, PQ for Platform Quality, WOM for Word of Mouth, UB for Usage Barrier, TB for Tradition Barrier, RB for Risk Barrier, SD for Standard Deviation.

**Table 3:** Heterotrait-Monotrait ratio (HTMT).

Constructs	ATT	COV	CV	EPV	INV	PQ	RB	SV	TB	UB
**COV**	0.857									
**CV**	0.656	0.522								
**EPV**	0.766	0.697	0.438							
**INV**	0.865	0.855	0.510	0.791						
**PQ**	0.753	0.606	0.393	0.731	0.724					
**RB**	0.055	0.059	0.077	0.063	0.056	0.052				
**SV**	0.858	0.655	0.464	0.727	0.721	0.596	0.045			
**TB**	0.863	0.696	0.502	0.665	0.729	0.613	0.056	0.808		
**UB**	0.319	0.187	0.202	0.187	0.189	0.234	0.192	0.150	0.264	
**WOM**	0.680	0.574	0.374	0.536	0.574	0.468	0.164	0.585	0.643	0.207

### 5.3. Structural model analysis

**Fig 2:**
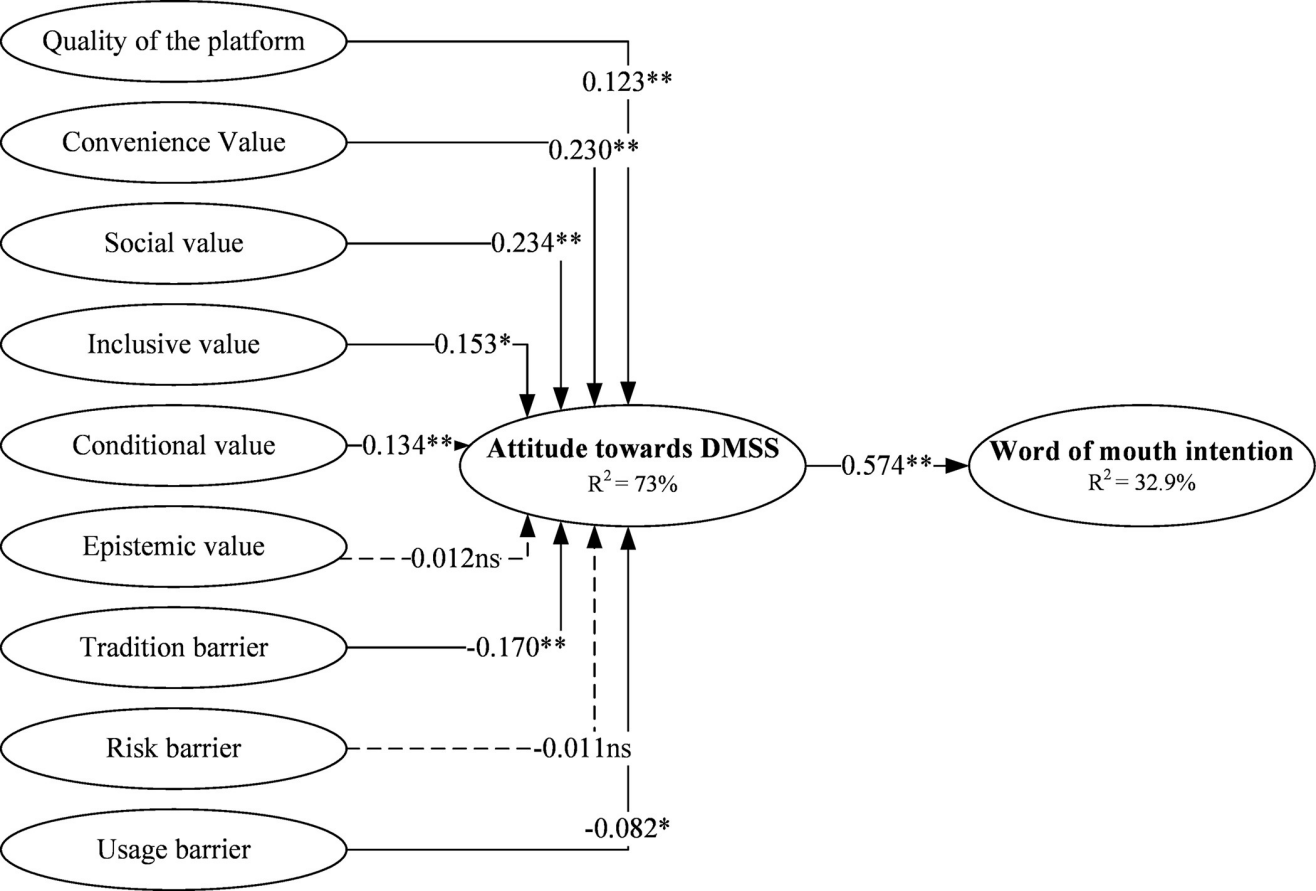
Results of PLS-SEM. ** = significance at p < 0.01, * = significance at p < 0.05, ns = not significant.

Hair, Hult [72] suggested that the research model path analysis was performed after the measurement model assessment. The hypothesized relationships have been tested using the bootstrapping method (5000 resamples) with a significance threshold of 0.05. The model accounted for 32.9 percent of the variance in user attitudes toward word-of-mouth and 73 percent of the variance in user attitudes toward DMSS adoption. Further, the Q^2^ value was measured to assess the model’s predictive validity [82]. The measured Q^2^ value in SEM must be higher than zero for a particular endogenous latent construct [83]. This study shows that Q^2^ for attitude is 0.437 and WOM is 0.238, which is greater than the recommended threshold. In a nutshell, among the ten proposed hypotheses we found eight hypotheses were supported as follows: PQ -> ATT (β = 0.123, t = 3.524); COV -> ATT (β = 0.230, t = 4.928); SV -> ATT (β = 0.234, t = 4.711); INV -> ATT (β = 0.153, t = 2.763); CV -> ATT (β = 0.134, t = 3.441); TB -> ATT (β = -0.170, t = 3.219); UB -> ATT (β = -0.082, t = 3.035); and ATT -> WOM (β = 0.574, t = 15.936). However, the results showed that two hypotheses were not statistically significant: EPV -> ATT (β = 0.012, t = 0.265) and RB -> ATT (β = -0.011, t = 0.297). The results of the hypotheses are shown in Table 4 and Fig 2.

**Table 4:** Hypothesis test.

Hypothesis	Path	*Coefficient (B)*	*t- statistics*	Supported	R^2^	F^2^	Q^2^	VIF
H1	PQ -> ATT	0.123	3.524	Yes	0.730	0.031	0.437	1.823
H2	COV -> ATT	0.230	4.928	Yes		0.08		2.446
H3	SV -> ATT	0.234	4.711	Yes		0.088		2.309
H4	INV -> ATT	0.153	2.763	Yes		0.027		3.195
H5	CV -> ATT	0.134	3.441	Yes		0.052		1.278
H6	EPV -> ATT	0.012	0.265	No		0		2.305
H7	TB -> ATT	-0.170	3.219	Yes		0.046		2.327
H8	RB -> ATT	-0.011	0.297	No		0		1.038
H9	UB -> ATT	-0.082	3.035	Yes		0.023		1.101
H10	ATT -> WOM	0.574	15.936	Yes	0.329	0.491	0.238	1.000

Note: Significant at p < 0.05.

## 6. Discussion

The results of our study shed light on the complex relationships between various factors that influence citizens’ initial adoption and post-adoption behavior of DMSS. Our analysis revealed several key enablers and barriers affecting users’ attitudes toward and intention to use the platform. First and foremost, it’s evident that quality and convenience are significant enablers for citizens looking to engage with DMSS. Users who perceive the e-government platform as high quality and easy to use are more likely to adopt it [10]. Social value and inclusiveness also emerged as significant factors that positively impact users’ attitudes toward DMSS [84]. These findings support previous research [8, 10] and indicate that individuals are more inclined to adopt DMSS if they perceive its use as compatible with their social identity. Furthe, the inclusion of diverse age groups, such as the elderly [17] and the younger population [85], on the platform also contributes to this likelihood.

According to the study, the conditional value significantly improves the public’s perception of DMSS by offering superior alternative services. The findings suggest that citizens have a more positive attitude toward DMSS when they consider reliability, security, and ease of use essential. This is especially true when DMSS allows them to complete transactions without needing to visit government offices in person, making them more likely to use DMSS [17, 44]. However, the findings revealed that epistemic value was unexpectedly not a significant predictor of the attitude toward the intention to use DMSS. This result aligns with earlier research conducted in China, which found that urban residents lacked the expertise to accurately recognize products with green certification [86]. Prior research also indicated epistemic value is not a significant predictor of consumers’ attitudes or intentions to adopt digital services, with utilitarian and economic factors playing a more prominent role [87, 88].

On the other hand, our study identified that both traditional and usage barriers negatively impact users’ attitudes toward DMSS [51, 52]. In other words, citizens who believe that DMSS goes against established norms and practices or find the platform difficult to use are less likely to adopt it. Bangladesh’s citizens may prefer traditional channels when accessing public services due to their past experiences [17]. This is consistent with earlier studies that suggested a relationship between prior behavior and intention to use new technologies [16, 56]. Surprisingly, the risk barrier did not significantly impact users’ attitudes and contradicted much earlier research [8, 61]. However, this result is also consistent with earlier research. For instance, earlier studies have shown that risk perception is not a significant predictor of technology acceptance and use [89, 90]. This result suggests that users may not perceive the DMSS platform as highly risky or uncertain. It could also indicate that the potential benefits of using DMSS outweigh any concerns regarding security or privacy. Likewise, if users are satisfied with the DMSS and their families and friends have a favorable opinion about using DMSS, it can be considered a safe way to obtain services. Therefore, the importance of perceived risk in this study was not a significant concern for Bangladesh’s citizens due to the abovementioned reasons [17].

Finally, the noteworthy influence of attitude toward behavioral intention to use DMSS on WOM intention highlights the crucial role of user perceptions in influencing their advocacy behaviors [25, 65]. This result relates to prior studies that underscore the impact of user attitudes on their intention to recommend technology-driven solutions [91]. When citizens have a positive attitude toward using DMSS, they are more likely to engage in favorable word-of-mouth (WOM) communication. This is often influenced by their satisfaction with the platform’s usability, functionality, and overall experience [24, 26]. This finding emphasizes the significance of establishing a positive user experience and cultivating positive attitudes toward the platform. Doing so can result in organic growth and heightened awareness of the platform.

### 6.1. Theoretical implications

This study offers several novel contributions to e-government and technology adoption research by applying an innovative approach through the SOR model. Specifically, it brings new insights into how facilitators and inhibitors concurrently shape user attitudes and behavior toward adopting DMSS. While previous studies have often focused on either enablers or barriers, our research integrates these factors into a single, unified framework, offering a more comprehensive understanding of e-government adoption.

An essential contribution of this study is integrating facilitators (enablers) and inhibitors (barriers) within a dual-factor model, addressing a significant gap in the existing literature. Previous research in e-government adoption focused on either enablers, such as perceived ease of use and usefulness, or inhibitors, such as resistance to change. Those studies did not explore their combined influence on user behavior. By incorporating both positive drivers through TCV and negative barriers through IRT into a single model, this study provides a more holistic and nuanced perspective on how users form attitudes toward e-government platforms [92]. This dual-process approach allows for a deeper understanding of users’ complex decision-making process, offering a more accurate reflection of real-world behavior than traditional models that focus solely on enablers [93].

In this study, TCV and IRT are crucial for understanding the enablers and inhibitors of e-government adoption, each offering unique theoretical insights. TCV suggests that consumer choices are influenced by different types of value, which are crucial in understanding how users perceive the benefits of services like DMSS. By applying TCV, this study shows how perceived convenience, social recognition, and knowledge shape users’ attitudes toward adopting DMSS. This framework helps explain why users find these services appealing, especially in public services, where value perceptions vary based on individual needs and priorities [93].

On the other hand, IRT explains the barriers to adoption by identifying sources of user resistance associated with new technologies. In the context of DMSS, IRT helps clarify why certain users may hesitate to transition from traditional government interactions to digital platforms. By examining inhibitors through IRT, this study offers insights into the psychological and cultural factors that slow down e-government adoption. This is more befitting in environments where users may feel uncomfortable or skeptical about technological change. TCV and IRT provide a comprehensive lens for understanding both the positive motivations and negative barriers shaping user behavior in adopting e-government platforms [92].

Another novel aspect of this study is emphasizing WOM intention as a key ‘Response’ variable. While previous studies have primarily focused on direct adoption or usage intention, we explore WOM as a critical driver for the broader diffusion of e-government services. In e-government research, WOM has been underexplored, but it can have a powerful impact on service uptake, as users’ recommendations can significantly influence others’ decisions to adopt digital services [94]. WOM also helps build trust, which is a key facilitator in adopting digital platforms [9]. This study, therefore, highlights the social influence of WOM and its potential to extend the reach of e-government platforms.

Although the SOR model has been used in various domains, its application to e-government adoption is still relatively rare. By applying the SOR framework in this context, we illustrate how external stimuli (enablers and inhibitors) influence internal states (attitudes) and lead to behavioral outcomes (WOM intention). This approach offers a more dynamic view of how users engage with e-government services, making it possible to better understand both the emotional and cognitive processes involved in adoption decisions [95]. WOM intention is not only an outcome of user satisfaction but also a mechanism through which e-government platforms can achieve long-term sustainability through network effects.

### 6.2. Practical implications

Our study provides valuable insights for policymakers creating and implementing e-government services. The findings suggest that the quality of the e-government platform, convenience value, social value, inclusiveness value, and conditional value all positively impact users’ attitudes toward adopting the DMSS. To achieve this, the government needs to invest in developing e-government systems that are user-friendly, accessible, secure, and reliable. Besides, continuous efforts should be made to improve the quality of these systems through regular maintenance and upgrading, user feedback, and system performance monitoring. This finding can also serve as a benchmark for e-government system providers and developers, who can use it to ensure that their systems meet the quality expectations of the citizens [10].

Specifically, convenience can be improved through user-friendly interfaces, easy access to information, and efficient service delivery processes [9]. Policymakers can use transparent and well-structured service designs to increase convenience and effectiveness in such services. Incorporating multiple channels of access to e-government services can also improve convenience and accessibility, positively impacting citizens’ attitudes [20, 68]. Furthermore, the findings suggest that policymakers should focus on the perception and reputation of e-government services among citizens to enhance the social value of these services. Policymakers can achieve this by creating positive social values of e-government services through marketing and public relations efforts. Ultimately, this will encourage citizens to adopt these services and increase their usage [8, 68].

Moreover, the finding emphasizes the significance of developing and executing inclusive e-government services that address all citizens’ various requirements and preferences when using such services. Collaborating with technology providers, user experience experts, and other stakeholders can assist policymakers in designing and implementing accessible, user-friendly, and inclusive services. E-government services must offer clear benefits to citizens and be user-friendly, convenient, and efficient to improve their perception of conditional value [44].

On the other hand, to overcome traditional and usage barriers, policymakers should invest in raising awareness and understanding of DMSS services through training and support for citizens. Regular consultations with citizens to understand their needs and preferences can also help overcome barriers to adopting the services. Improving citizens’ digital literacy and awareness through education can reduce barriers to usage. Providing user-friendly interfaces and adequate technical support can promote inclusivity and empower citizens to engage in e-government services [17, 44]. Finally, the positive relationship between user attitude and word-of-mouth intention highlights the importance of encouraging users to perceive e-government services positively. Policymakers should prioritize investing in communication and marketing strategies that enhance awareness and understanding of e-government services. These strategies should also aim to promote the benefits of these services, encouraging users to speak positively about them and encourage others to utilize them [8, 68].

## 7. Limitations and future research directions

It is essential to recognize the limitations of the current study. Firstly, the study was limited in generalizing to other people or locations because it was conducted in a particular community in Bangladesh. Secondly, the data was collected through self-administered questionnaires, which may have introduced a social desirability bias in the responses. Given these limitations, there are several areas for future research to further build on the insights gained from this study. Future studies could extend the present research to different populations and regions to examine the generalizability of the findings. In addition, it would be valuable to collect data using more objective measures, such as usage logs, to overcome the limitations of self-reported measures. Thirdly, future studies should consider additional elements that can affect people’s attitudes regarding e-government services, such as sociodemographic traits, prior technology-related experience, and perceptions of privacy and security. Finally, it would be helpful to study the impact of policy interventions designed to address usage barriers on citizens’ attitudes toward adopting e-government services.

## 8. Conclusion

In conclusion, the present study aimed to shed light on the drivers and hindrances of citizens’ acceptance and utilization of DMSS, a one-stop e-government service platform. The results showed that the quality of the platform, convenience value, social value, and inclusiveness value were the critical drivers for DMSS adoption. On the other hand, the traditional and usage barriers were identified as the main barriers that hindered citizens from using the platform. The study also revealed that users’ attitude significantly affects their word-of-mouth intention towards the DMSS, emphasizing improving citizens’ perception of the platform. These findings hold significant implications for policymakers in developing and implementing e-government services. By addressing the barriers and enhancing the drivers, policymakers can increase the likelihood of citizens’ adoption and successful implementation of e-government services. Future research can provide a more detailed exploration of the factors and their impact on e-government service adoption in diverse contexts and countries.

## Supporting information

S1 Appendix Measurement items. (DOCX)

S2 Appendix Demographic profile of the respondents. (DOCX)

S1 Data Dataset_PLOS ONE. (XLSX)

S1 Questionnaire Survey questionnaire. (DOCX)

## References

[1] Ilieva G, Yankova T, Ruseva M, Dzhabarova Y, Zhekova V, Klisarova-Belcheva S, et al. Factors Influencing User Perception and Adoption of E-Government Services. Administrative Sciences [Internet]. 2024; 14(3).

[2] Hossain MN, Talukder MS, Hoque MR, Bao Y. The use of open government data to citizen empowerment: an empirical validation of a proposed model. Foresight. 2018;20(6):665–80.

[3] Yera A, Arbelaitz O, Jauregui O, Muguerza J. Characterization of e-Government adoption in Europe. PLoS One. 2020;15(4):e0231585. doi: 10.1371/journal.pone.0231585 32302326 PMC7164598

[4] Liu L, Ju J, Feng Y. An extensible framework for collaborative e-governance platform workflow modeling using data flow analysis. Information Technology for Development. 2017;23(3):415–37.

[5] Biswas B, Roy SK. Service quality, satisfaction and intention to use Union Digital Center in Bangladesh: The moderating effect of citizen participation. PLoS One. 2021;15(12):e0244609. 10.1371/journal.pone.0244609 PMC7769423 33370421

[6] Ramirez-Madrid JP, Escobar-Sierra M, Lans-Vargas I, Montes Hincapie JM. Factors influencing citizens’ adoption of e-government: an empirical validation in a Developing Latin American Country. Public Management Review. 2024;26(1):185–218.

[7] AbdulKareem AK, Oladimeji KA. Cultivating the digital citizen: trust, digital literacy and e-government adoption. Transforming Government: People, Process and Policy. 2024;18(2):270–86.

[8] Dwivedi YK, Rana NP, Janssen M, Lal B, Williams MD, Clement M. An empirical validation of a unified model of electronic government adoption (UMEGA). Government Information Quarterly. 2017;34(2):211–30.

[9] Mensah IK. Impact of Government Capacity and E-Government Performance on the Adoption of E-Government Services. International Journal of Public Administration. 2020;43(4):303–11.

[10] Li Y, Shang H. Service quality, perceived value, and citizens’ continuous-use intention regarding e-government: Empirical evidence from China. Information & Management. 2020;57(3):103197.

[11] Hasan A, Alenazy AA, Habib S, Husain S. Examining the drivers and barriers to adoption of e-government services in Saudi Arabia. Journal of Innovative Digital Transformation. 2024;ahead-of-print(ahead-of-print).

[12] Saleh M. E-government Services in Libya: User Perception and Adoption Barriers. Journal of Reproducible Research. 2024;2(2):19–25.

[13] Gupta KP, Bhaskar P, Bhaskar P. Inhibiting and enabling factors influencing employees’ adoption of e-government: prioritisation using analytic hierarchy process. Electronic Government, an International Journal. 2023;19(6):641–66.

[14] Sabani A, Thai V, Hossain MA. Factors Affecting Citizen Adoption of E-Government in Developing Countries: An Exploratory Case Study From Indonesia. Journal of Global Information Management (JGIM). 2023;31(1):1–23.

[15] Rana NP, Dwivedi YK, Williams MD, Lal B. Examining the success of the online public grievance redressal systems: an extension of the IS success model. Information Systems Management. 2015;32(1):39–59.

[16] Talukder MS, Laato S, Islam AKMN, Bao Y. Continued use intention of wearable health technologies among the elderly: an enablers and inhibitors perspective. Internet Research. 2021;ahead-of-print(ahead-of-print).

[17] Talukder S, Chiong R, Corbitt B, Bao Y. Critical Factors Influencing the Intention to Adopt M-Government Services by the Elderly. Journal of Global Information Management. 2020;28(4):419–38.

[18] Mehrabian A, Russell JA. An approach to environmental psychology. Cambridge, MA, US: The MIT Press; 1974. xii, 266–xii, p.

[19] Davis F. Perceived usefulness, perceived ease of use, and user acceptance of information technology. MIS Quarterly. 1989;13(3):319–40.

[20] Al-Hujran O, Al-Debei MM, Chatfield A, Migdadi M. The imperative of influencing citizen attitude toward e-government adoption and use. Computers in Human Behavior. 2015;53:189–203.

[21] Herzberg FI. Work and the nature of man. Oxford, England: World; 1966.

[22] Sheth JN, Newman BI, Gross BL. Why we buy what we buy: A theory of consumption values. Journal of Business Research. 1991;22(2):159–70.

[23] Liu Y, Li H, Kostakos V, Goncalves J, Hosio S, Hu F. An empirical investigation of mobile government adoption in rural China: A case study in Zhejiang province. Government Information Quarterly. 2014;31(3):432–42.

[24] Alghamdi SY, Kaur S, Qureshi KM, Almuflih AS, Almakayeel N, Alsulamy S, et al. Antecedents for online food delivery platform leading to continuance usage intention via e-word-of-mouth review adoption. PLoS One. 2023;18(8):e0290247. doi: 10.1371/journal.pone.0290247 37590240 PMC10434875

[25] Yasir A, Hu X, Ahmad M, Rauf A, Shi J, Ali Nasir S. Modeling Impact of Word of Mouth and E-Government on Online Social Presence during COVID-19 Outbreak: A Multi-Mediation Approach. International Journal of Environmental Research and Public Health [Internet]. 2020; 17(8). doi: 10.3390/ijerph17082954 32344770 PMC7216275

[26] Potnis D, Gala B. Factors Influencing Electronic Word-of-Mouth Among Indian Youth: Implications for Mobile Governance. Proceedings of the Special Collection on eGovernment Innovations in India; New Delhi AA, India: Association for Computing Machinery; 2017. p. 107–14.

[27] Malodia S, Dhir A, Mishra M, Bhatti ZA. Future of e-Government: An integrated conceptual framework. Technological Forecasting and Social Change. 2021;173:121102.

[28] Ebrahim Z, Irani Z. E‐government adoption: architecture and barriers. Business Process Management Journal. 2005;11(5):589–611.

[29] Jacob DW, Fudzee MFM, Salamat MA, Herawan T. A review of the generic end-user adoption of e-government services. International Review of Administrative Sciences. 2019;85(4):799–818.

[30] Rana NP, Dwivedi YK. Citizen’s adoption of an e-government system: Validating extended social cognitive theory (SCT). Government Information Quarterly. 2015;32(2):172–81.

[31] Rey-Moreno M, Felício JA, Medina-Molina C, Rufín R. Facilitator and inhibitor factors: Adopting e-government in a dual model. Journal of Business Research. 2018;88:542–9.

[32] Jacoby J. Stimulus-Organism-Response Reconsidered: An Evolutionary Step in Modeling (Consumer) Behavior. Journal of Consumer Psychology. 2002;12(1):51–7.

[33] Zhu L, Li H, Wang F-K, He W, Tian Z. How online reviews affect purchase intention: a new model based on the stimulus-organism-response (—) framework. Aslib Journal of Information Management. 2020;72(4):463–88.

[34] Fan X, Ning N, Deng N. The impact of the quality of intelligent experience on smart retail engagement. Marketing Intelligence & Planning. 2020;38(7):877–91.

[35] Tak P, Gupta M. Examining Travel Mobile App Attributes and Its Impact on Consumer Engagement: An Application of S-O-R Framework. Journal of Internet Commerce. 2021;20(3):293–318.

[36] Chopdar PK, Balakrishnan J. Consumers response towards mobile commerce applications: S-O-R approach. International Journal of Information Management. 2020;53:102106.

[37] Palash MAS, Talukder MS, Islam AKMN, Bao Y. Positive and negative valences, personal innovativeness and intention to use facial recognition for payments. Industrial Management & Data Systems. 2022;122(4):1081–108.

[38] Najmul Islam AKM, Cenfetelli R, Benbasat I. Organizational buyers’ assimilation of B2B platforms: Effects of IT-enabled service functionality. The Journal of Strategic Information Systems. 2020;29(1):101597.

[39] Baumeister RF, Bratslavsky E, Finkenauer C, Vohs KD. Bad is Stronger than Good. Review of General Psychology. 2001;5(4):323–70.

[40] Cenfetelli RT. Inhibitors and enablers as dual factor concepts in technology usage. Journal of the Association for Information Systems. 2004;5(11):16.

[41] Kaur P, Dhir A, Talwar S, Ghuman K. The value proposition of food delivery apps from the perspective of theory of consumption value. International Journal of Contemporary Hospitality Management. 2021;33(4):1129–59.

[42] Tandon A, Kaur P, Bhatt Y, Mäntymäki M, Dhir A. Why do people purchase from food delivery apps? A consumer value perspective. Journal of Retailing and Consumer Services. 2021;63:102667.

[43] Talwar S, Dhir A, Kaur P, Mäntymäki M. Why do people purchase from online travel agencies (OTAs)? A consumption values perspective. International Journal of Hospitality Management. 2020;88:102534.

[44] Rahman F, Talukder S, Lanrong Y. Enablers and inhibitors of e-tax system use: the perspective of dual-factor concepts. International Journal of Managing Public Sector Information and Communication Technologies. 2021;12(1).

[45] Hsu F-M, Chen T-Y. Understanding information systems usage behavior in E-Government: The role of context and perceived value. PACIS 2007 proceedings. 2007:41.

[46] Lin P-C, Huang Y-H. The influence factors on choice behavior regarding green products based on the theory of consumption values. Journal of Cleaner Production. 2012;22(1):11–8.

[47] Hornung H, Baranauskas MCC. Towards a design rationale for inclusive eGovernment services. International Journal of Electronic Government Research (IJEGR). 2011;7(3):1–20.

[48] Wang H-Y, Liao C, Yang L-H. What affects mobile application use? The roles of consumption values. International Journal of Marketing Studies. 2013;5(2):11.

[49] Savoldelli A, Codagnone C, Misuraca G. Understanding the e-government paradox: Learning from literature and practice on barriers to adoption. Government Information Quarterly. 2014;31:S63–S71.

[50] Choe JY, Kim S. Effects of tourists’ local food consumption value on attitude, food destination image, and behavioral intention. International Journal of Hospitality Management. 2018;71:1–10.

[51] Seth H, Talwar S, Bhatia A, Saxena A, Dhir A. Consumer resistance and inertia of retail investors: Development of the resistance adoption inertia continuance (RAIC) framework. Journal of Retailing and Consumer Services. 2020;55:102071.

[52] Kaur P, Dhir A, Singh N, Sahu G, Almotairi M. An innovation resistance theory perspective on mobile payment solutions. Journal of Retailing and Consumer Services. 2020;55:102059.

[53] Kushwah S, Dhir A, Sagar M, Gupta B. Determinants of organic food consumption. A systematic literature review on motives and barriers. Appetite. 2019;143:104402. doi: 10.1016/j.appet.2019.104402 31421197

[54] Samuelson W, Zeckhauser R. Status quo bias in decision making. Journal of Risk and Uncertainty. 1988;1(1):7–59.

[55] Polites GL, Karahanna E. Shackled to the Status Quo: The Inhibiting Effects of Incumbent System Habit, Switching Costs, and Inertia on New System Acceptance. MIS Quarterly. 2012;36(1):21–42.

[56] Hoque R, Sorwar G. Understanding factors influencing the adoption of mHealth by the elderly: An extension of the UTAUT model. International Journal of Medical Informatics. 2017;101:75–84. doi: 10.1016/j.ijmedinf.2017.02.002 28347450

[57] Bauer RA. Consumer Behavior as Risk Taking. Proceedings of the 43rd National Conference of the American Marketing Assocation, June 15, 16, 17, Chicago, Illinois, 1960. 1960.

[58] Saxena S. Role of “perceived risks” in adopting mobile government (m-government) services in India. foresight. 2018(just-accepted):00-.

[59] Schaupp LC, Carter L, McBride ME. E-file adoption: A study of U.S. taxpayers’ intentions. Computers in Human Behavior. 2010;26(4):636–44.

[60] Ram S, Sheth JN. Consumer Resistance to Innovations: The Marketing Problem and its solutions. Journal of Consumer Marketing. 1989;6(2):5–14.

[61] Talukder MS, Sorwar G, Bao Y, Ahmed JU, Palash MAS. Predicting antecedents of wearable healthcare technology acceptance by elderly: A combined SEM-Neural Network approach. Technological Forecasting and Social Change. 2020;150:119793.

[62] Ajzen I. The theory of planned behavior. Organizational behavior and human decision processes. 1991;50(2):179–211.

[63] Rahi S. Assessing individual behavior towards adoption of telemedicine application during COVID-19 pandemic: evidence from emerging market. Library Hi Tech. 2022;40(2):394–420.

[64] Godes D, Mayzlin D. Using online conversations to study word-of-mouth communication. Marketing science. 2004;23(4):545–60.

[65] Wang H-C, Doong H-S. Does government effort or citizen word-of-mouth determine e-Government service diffusion? Behaviour & Information Technology. 2010;29(4):415–22.

[66] Gelb B, Johnson M. Word-of-mouth communication: Causes and consequences. Journal of Marketing Health Services. 1995;15(3):54. 10152795

[67] Rahi S. Research design and methods: A systematic review of research paradigms, sampling issues and instruments development. International Journal of Economics Management Sciences. 2017;6(2):1–5.

[68] Talukder S, Chiong R, Dhakal S, Sorwar G, Bao Y. A two-stage structural equation modeling-neural network approach for understanding and predicting the determinants of m-government service adoption. Journal of Systems and Information Technology. 2019;21(4):419–38.

[69] Patton MQ. Qualitative research and evaluation methods. Thousand Oaks. Cal: Sage Publications. 2002;4.

[70] Creswell JW, Creswell JD. Research design: Qualitative, quantitative, and mixed methods approaches: Sage publications; 2017.

[71] Cheff R. Compensating research participants: A survey of current practices in Toronto: Wellesley Institute; 2018.

[72] Hair JF, Hult GTM, Ringle C, Sarstedt M. A primer on partial least squares structural equation modeling (PLS-SEM). Thousand Oaks, CA, USA.: SaGe Publications; 2016.

[73] Faul F, Erdfelder E, Lang A-G, Buchner A. G*Power 3: A flexible statistical power analysis program for the social, behavioral, and biomedical sciences. Behavior Research Methods. 2007;39(2):175–91. doi: 10.3758/bf03193146 17695343

[74] Joseph F, Black WC, Babin BJ, Anderson RE. Multivariate data analysis: A global perspective: Pearson Education; 2010.

[75] Fornell C, Larcker DF. Evaluating Structural Equation Models with Unobservable Variables and Measurement Error. Journal of Marketing Research. 1981;18(1):39–50.

[76] Hair JF, Sarstedt M, Ringle CM, Gudergan SP. Advanced issues in partial least squares structural equation modeling. Thousand Oaks, CA, USA: SaGe Publications; 2017.

[77] Anderson JC, Gerbing DW. Structural equation modeling in practice: A review and recommended two-step approach. Psychological Bulletin. 1988;103(3):411–23.

[78] Podsakoff PM, MacKenzie SB, Lee J-Y, Podsakoff NP. Common method biases in behavioral research: A critical review of the literature and recommended remedies. Journal of applied psychology. 2003;88(5):879. doi: 10.1037/0021-9010.88.5.879 14516251

[79] Malhotra NK, Kim SS, Patil A. Common Method Variance in IS Research: A Comparison of Alternative Approaches and a Reanalysis of Past Research. Management Science. 2006;52(12):1865–83.

[80] Shmueli G, Sarstedt M, Hair Joseph F, Cheah J-H, Ting H, Vaithilingam S, et al. Predictive model assessment in PLS-SEM: guidelines for using PLSpredict. European Journal of Marketing. 2019;53(11):2322–47.

[81] Henseler J, Ringle CM, Sarstedt M. A new criterion for assessing discriminant validity in variance-based structural equation modeling. Journal of the Academy of Marketing Science. 2015;43(1):115–35.

[82] Stone M. Cross-validatory choice and assessment of statistical predictions. Journal of the royal statistical society Series B (Methodological). 1974;36(2):111–47.

[83] Tenenhaus M, Vinzi VE, Chatelin Y-M, Lauro C. PLS path modeling. Computational Statistics & Data Analysis. 2005;48(1):159–205.

[84] Dholakia UM, Bagozzi RP, Pearo LK. A social influence model of consumer participation in network- and small-group-based virtual communities. International Journal of Research in Marketing. 2004;21(3):241–63.

[85] Wang C. Antecedents and consequences of perceived value in Mobile Government continuance use: An empirical research in China. Computers in Human Behavior. 2014;34:140–7.

[86] Awuni JA, Du J. Sustainable Consumption in Chinese Cities: Green Purchasing Intentions of Young Adults Based on the Theory of Consumption Values. Sustainable Development. 2016;24(2):124–35.

[87] Ståhl T, Turner J. Epistemic values and the Big Five: Personality characteristics of those who ascribe personal and moral value to epistemic rationality. PLoS One. 2021;16(10):e0258228. doi: 10.1371/journal.pone.0258228 34610048 PMC8491882

[88] Al-Debei MM, Al-Lozi E. Explaining and predicting the adoption intention of mobile data services: A value-based approach. Computers in Human Behavior. 2014;35:326–38.

[89] Veeramootoo N, Nunkoo R, Dwivedi YK. What determines success of an e-government service? Validation of an integrative model of e-filing continuance usage. Government Information Quarterly. 2018;35(2):161–74.

[90] Rahman MF, Talukder MS, Lanrong Y, Khayer A. Why do citizens use e-tax system? Extending the technology continuance theory. International Journal of Research in Business and Social Science (2147–4478). 2020;9(7):177–89.

[91] Acharya A. The impact of brand familiarity, customer brand engagement and self-identification on word-of-mouth. South Asian Journal of Business Studies. 2021;10(1):29–48.

[92] Lallmahomed MZI, Lallmahomed N, Lallmahomed GM. Factors influencing the adoption of e-Government services in Mauritius. Telematics and Informatics. 2017;34(4):57–72.

[93] Amaya Rivas A, Liao Y-K, Vu M-Q, Hung C-S. Toward a Comprehensive Model of Green Marketing and Innovative Green Adoption: Application of a Stimulus-Organism-Response Model. Sustainability [Internet]. 2022; 14(6).

[94] Rana NP, Dwivedi YK, Lal B, Williams MD, Clement M. Citizens’ adoption of an electronic government system: towards a unified view. Information Systems Frontiers. 2017;19(3):549–68.

[95] Ziba PW, Kang J. Factors affecting the intention to adopt e-government services in Malawi and the role played by donors. Information Development. 2019;36(3):369–89.

