# Supplementary material for: Facilitators and inhibitors of attitude and word-of-mouth intention toward adoption of digital municipal service systems: A stimulus-organism-response approach
Source: PLoS One. 2024 Dec 18;19(12):e0315009. doi: 10.1371/journal.pone.0315009 (PMC11654987; doi:10.1371/journal.pone.0315009)
Supplement: S1 Appendix — (DOCX) [file pone.0315009.s001.docx]

**S1 Appendix A. Measurement Items.**

| **Construct** | **Corresponding Items** | **Items Sources** |
| --- | --- | --- |
| Quality Value | QV1. The digital municipal service system (DMSS) has consistent quality  QV2. The DMSS has is well developed.  QV3. The DMSS has an acceptable standard of quality  QV4. The DMSS would perform consistently | Lin and Huang (1) and Sheth, Newman (2) |
| Social Value | SV1. Using the DMSS has improved the way others perceive me  SV2. The DMSS is used by many people that I know  SV3. Using the DMSS would make a good impression on other people | Lin and Huang (1) and Sheth, Newman (2) |
| Epistemic Value | EPV1. I would use the DMSS to test new way of doing things  EPV2. I would use the DMSS to try new technologies  EPV3. I would use the DMSS out of curiosity | Lin and Huang (1) and Sheth, Newman (2) |
| Conditional Value | CV1. I would use the DMSS platform instead of conventional method when the service that I want is available in the electronic platform.  CV2. I prefer to use the DMSS platform instead of conventional method when the service that I want is available in the electronic platform.  CV3. I would use the DMSS platform instead of conventional method if there were a subsidy for digital municipal service system. | Lin and Huang (1) and Sheth, Newman (2) |
| Convenience Value | COV1. I save time when I transact with the DMSS platform.  COV2. I value the ease of using the DMSS platform.  COV3. Using the DMSS platform makes my life easier | Li and Shang (3) |
| Inclusiveness Value | INV1. Using the DMSS makes the public service more available to more people.  INV2. Using the DMSS is valuable to providing equal public service to all citizens.  INV3. Using the DMSS makes the disadvantaged groups benefit more from public service | Li and Shang (3) |
| Usage barrier (UB) | UB1. In my opinion, the use of DMSS is inconvenient.  UB2. To my knowledge, DMSS is not easy to use.  UB3. I think that DMSS is not fast to use.  UB4. In my opinion, progress in DMSS is not clear. | Laukkanen (4) |
| Image Barrier (IB) | IB1: In my opinion, DMSS is often too complicated to be useful.  IB2: I have such an image that DMSS is difficult to use. | Laukkanen (4) |
| Risk barrier | RB1: It is probable that DMSS would frustrate me because of its poor performance.  RB2: Comparing with other technologies, using DMSS has more uncertainties.  RB3: It is uncertain whether DMSS would be as effective as I think.  RB4: I fear that while I am using an DMSS, someone may hack my account. | Laukkanen (4) |
| Tradition Barrier (TB) | TB1: I would not comply with change to the new way of working with DMSS.  TB2: I will not cooperate with the change to the new way of working with the DMSS.  TB3: I oppose the change to the new way of working with the new way of working with DMSS.  TB4: I do not agree with the change to the new way of working with the DMSS. | Laukkanen (4) |
| Attitude (ATT) | ATT1: Using the DMSS to access government services is a good idea.  ATT2: I like the use of DMSS to access government services  ATT3: Using the DMSS to access government services would be pleasant | Al-Hujran, Al-Debei (5) |
| Word of Mouth (WOM) | WOM1. I would recommend DMSS to others.  WOM2. I would tell my friends and family about my experience with DMSS.  WOM3. I would encourage others to use DMSS. | Kang, Hong (6) and Potnis and Gala (7) |
