# Supplementary material for: Facilitators and inhibitors of attitude and word-of-mouth intention toward adoption of digital municipal service systems: A stimulus-organism-response approach
Source: PLoS One. 2024 Dec 18;19(12):e0315009. doi: 10.1371/journal.pone.0315009 (PMC11654987; doi:10.1371/journal.pone.0315009)
Supplement: S2 Appendix — (DOCX) [file pone.0315009.s002.docx]

**S2 Appendix B. Demographic profile of the respondents.**

| **Category** | **Demographic** | **Frequency** | **Percentage** |
| --- | --- | --- | --- |
| Gender | Male | 189 | 53.54% |
|  | Female | 164 | 46.46% |
| Age (Years) | 18-25 | 117 | 33.14% |
|  | 26-35 | 108 | 30.59% |
|  | 36-45 | 111 | 31.44% |
|  | 46-55 | 15 | 4.25% |
|  | 56 and above | 2 | 0.57% |
| Education | High school | 25 | 7.08% |
|  | Bachelor's degree | 205 | 58.07% |
|  | Master's degree | 123 | 34.84% |
|  | Doctoral degree | 0 | 0.00% |
| E-government service usage experience | Less than a year | 148 | 41.93% |
|  | 1-2 years | 143 | 40.51% |
|  | 2-3 years | 44 | 12.46% |
|  | More than 3 years | 18 | 5.10% |
| Region | Urban | 189 | 53.54% |
|  | Semi-Urban | 164 | 46.46% |
