# Supplementary material for: Facilitators and inhibitors of attitude and word-of-mouth intention toward adoption of digital municipal service systems: A stimulus-organism-response approach
Source: PLoS One. 2024 Dec 18;19(12):e0315009. doi: 10.1371/journal.pone.0315009 (PMC11654987; doi:10.1371/journal.pone.0315009)
Supplement: S1 Questionnaire — (DOCX) [file pone.0315009.s004.docx]

Dear Respondents,

I am asking your kind participation in our research study titled ***“Facilitators and Inhibitors of Digital Municipal Service System (DMSS) Using Behavior in Emerging Economics: The Case of Bangladesh”.*** This study aims to explore the factors influencing citizens' adoption of the Digital Municipal Service System (DMSS) in Bangladesh. Your participation in this survey will be highly appreciated and will immensely contribute to the research findings. We assured, your participation is voluntary, and you have the right to withdraw at any point. Additionally, to express our gratitude, we offer a token of appreciation for your valuable time and input.

We also assure that, your participation in this study will remain anonymous and your responses will be kept confidential and will exclusively be used for conducting research in a segregated manner. Your individual response, identity, organizational details will not be made public under any circumstances.

If you have any questions or concern, please contact at the below mentioned email addresses.

Regards,

| Dr. Md. Shamim Talukder  Assistant Professor, Department of Management,  School of Business & Economics, North South University  Email: shamim.talukder@northsouth.edu | Quazi Tafsirul Islam  Senior Lecturer of Strategy, Management & HR, Department of Management,  School of Business & Economics, North South University  Email: quazi.islam@northsouth.edu |
| --- | --- |

Thank you for your interest in the survey and the permission to use your valuable inputs.

**Statement of Consent:** By clicking "I agree" below, you are indicating that you have read and understood the information provided in this consent form, and that you voluntarily agree to participate in the study.

[ ] I agree

[ ] I do not agree

**Questionnaire**

For most questions simply circle the number or tick in the square that corresponds to your answer.

1. Gender

1. Female
2. Male

2. Age

1. 18–29 years
2. 30–39 years
3. 40–49 years
4. 50–59 years
5. >60 years

3. Education level

1. Below Secondary
2. Secondary
3. Bachelor degree
4. Post graduate

4. E-government services usages experience

1. Less than 3 years
2. 3 – 6 years
3. 7- 10 years
4. More than 10 years

5. Region

1. Urban
2. Semi-Urban
3. Rural

6. Division

1. Barishal Division
2. Chattogram Division
3. Dhaka Division
4. Khulna Division
5. Mymensingh Division
6. Rajshahi Division
7. Rangpur Division
8. Sylhet Division

**Please rate the degree to which you agree with the following statement.**

7 for Strongly Agree, 6 for Agree, 5 for Somewhat Agree, 4 for Neutral, 3 for Somewhat Disagree, 2 for Disagree, 1 for Strongly Disagree

| **Statement** | | | | | | | |
| --- | --- | --- | --- | --- | --- | --- | --- |
| QV1. The DMSS has consistent quality to access government services. | 1 | 2 | 3 | 4 | 5 | 6 | 7 |
| QV2. The DMSS has is well made to access government services. | 1 | 2 | 3 | 4 | 5 | 6 | 7 |
| QV3. The DMSS has an acceptable standard of quality to access government services. | 1 | 2 | 3 | 4 | 5 | 6 | 7 |
| QV4. The DMSS would perform consistently to access government services. | 1 | 2 | 3 | 4 | 5 | 6 | 7 |
| SV1. Using the DMSS has improved the way others perceive me to access government services. | 1 | 2 | 3 | 4 | 5 | 6 | 7 |
| SV2. The DMSS is used by many people that I know to access government services. | 1 | 2 | 3 | 4 | 5 | 6 | 7 |
| SV3. Using the DMSS would make a good impression on other people | 1 | 2 | 3 | 4 | 5 | 6 | 7 |
| EPV1. I would use the DMSS to test new way of doing things | 1 | 2 | 3 | 4 | 5 | 6 | 7 |
| EPV2. I would use the DMSS to try new technologies | 1 | 2 | 3 | 4 | 5 | 6 | 7 |
| EPV3. I would use the DMSS out of curiosity | 1 | 2 | 3 | 4 | 5 | 6 | 7 |
| CV1. I would use the DMSS platform instead of conventional method when the service that I want is available in the electronic platform. | 1 | 2 | 3 | 4 | 5 | 6 | 7 |
| CV2. I prefer to use the DMSS platform instead of conventional method when the service that I want is available in the electronic platform. | 1 | 2 | 3 | 4 | 5 | 6 | 7 |
| CV3. I would use the DMSS platform instead of conventional method if there were a subsidy for DMSS. | 1 | 2 | 3 | 4 | 5 | 6 | 7 |
| COV1. I save time when I transact with the DMSS platform. | 1 | 2 | 3 | 4 | 5 | 6 | 7 |
| COV2. I value the ease of using the DMSS platform. | 1 | 2 | 3 | 4 | 5 | 6 | 7 |
| COV3. Using the DMSS platform makes my life easier | 1 | 2 | 3 | 4 | 5 | 6 | 7 |
| INV1. Using the DMSS makes the public service more available to more people. | 1 | 2 | 3 | 4 | 5 | 6 | 7 |
| INV2. Using the DMSS is valuable to providing equal public service to all citizens. | 1 | 2 | 3 | 4 | 5 | 6 | 7 |
| INV3. Using the DMSS makes the disadvantaged groups benefit more from public service | 1 | 2 | 3 | 4 | 5 | 6 | 7 |
| UB1. In my opinion, the use of DMSS is inconvenient to access government services. | 1 | 2 | 3 | 4 | 5 | 6 | 7 |
| UB2. To my knowledge, DMSS is not easy to use. | 1 | 2 | 3 | 4 | 5 | 6 | 7 |
| UB3. I think that DMSS is not fast to use. | 1 | 2 | 3 | 4 | 5 | 6 | 7 |
| UB4. In my opinion, progress in DMSS is not clear. | 1 | 2 | 3 | 4 | 5 | 6 | 7 |
| RB1: It is probable that DMSS would frustrate me because of its poor performance. | 1 | 2 | 3 | 4 | 5 | 6 | 7 |
| RB2: Comparing with other technologies, using the DMSS has more uncertainties. | 1 | 2 | 3 | 4 | 5 | 6 | 7 |
| RB3: It is uncertain whether DMSS would be as effective as I think. | 1 | 2 | 3 | 4 | 5 | 6 | 7 |
| TB1: I would not comply with change to the new way of working with DMSS. | 1 | 2 | 3 | 4 | 5 | 6 | 7 |
| TB2: I will not cooperate with the change to the new way of working with the DMSS. | 1 | 2 | 3 | 4 | 5 | 6 | 7 |
| TB3: I oppose the change to the new way of working with DMSS to access government services. | 1 | 2 | 3 | 4 | 5 | 6 | 7 |
| TB4: I do not agree with the change to the new way of working with the DMSS. | 1 | 2 | 3 | 4 | 5 | 6 | 7 |
| ATT1: Using the DMSS to access government services is a good idea. | 1 | 2 | 3 | 4 | 5 | 6 | 7 |
| ATT2: I like to use the DMSS to access government services. | 1 | 2 | 3 | 4 | 5 | 6 | 7 |
| ATT3: Using the DMSS to access government services would be pleasant. | 1 | 2 | 3 | 4 | 5 | 6 | 7 |
| UI1: I expect my use of DMSS will increase in the future. | 1 | 2 | 3 | 4 | 5 | 6 | 7 |
| UI2: I intend to use the DMSS in the future to access government services. | 1 | 2 | 3 | 4 | 5 | 6 | 7 |
| UI3: If I have an opportunity, I will use the DMSS to access government services | 1 | 2 | 3 | 4 | 5 | 6 | 7 |
| UI4: I will always try to use the DMSS to access government services | 1 | 2 | 3 | 4 | 5 | 6 | 7 |
| WOM1: I will recommend the DMSS to my friends or colleagues to access government services | 1 | 2 | 3 | 4 | 5 | 6 | 7 |
| WOM2: If I have a good experience with the DMSS, I will recommend it to my friends. | 1 | 2 | 3 | 4 | 5 | 6 | 7 |
| WOM3. I will post or share a review about DMSS on social media or other online platforms to access government services. | 1 | 2 | 3 | 4 | 5 | 6 | 7 |
